# Design and evaluation of rufinamide nanocrystals loaded thermoresponsive nasal *in situ* gelling system for improved drug distribution to brain

**DOI:** 10.3389/fphar.2022.943772

**Published:** 2022-10-04

**Authors:** Avantika Dalvi, Punna Rao Ravi, Chandra Teja Uppuluri

**Affiliations:** Department of Pharmacy, BITS Pilani Hyderabad Campus, Hyderabad, India

**Keywords:** rufinamide, nanocrystals, thermoresponsive gel, nose-to-brain delivery, pharmacokinetics

## Abstract

Rufinamide (Rufi) is an antiepileptic drug used to manage Lennox-Gastaut Syndrome and partial seizures. The oral bioavailability of Rufi is less due to its poor solubility and low dissolution rate in the gastrointestinal fluids. This results in less amount of drug reaching the brain following the oral administration of drug. Oral formulations of Rufi are prescribed at a high dose and dosing frequency to increase its distribution to the brain. A Rufi loaded thermoresponsive nasal *in situ* gel which showed significantly high brain concentrations compared to aqueous suspension of Rufi administered through nasal route was developed by our research group and published. In the current work, we have formulated nanocrystals of Rufi and suspended them in a xyloglucan based thermoresponsive gel to improve the nose-to-brain distribution. The particle size, polydispersity index, and yield (%) of the optimized Rufi nanocrystals were 261.2 ± 2.1 nm, 0.28 ± 0.08, and 89.6 ± 2.0 respectively. The narrow PDI indicates that the manufacturing process is reproducible and reliable. Higher % yield suggested that the method of preparation is efficient. The sol-to-gel transition of *in situ* gel loaded with Rufi nanocrystals was at 32°C which suggested that the formulation transforms into gel at nasal epithelial temperatures. The nasal pharmacokinetic studies showed that Rufi nanocrystals loaded *in situ* gel produced higher concentration of the drug in brain (higher brain C_max_) and maintained the drug concentrations for longer duration (higher mean residence time) compared to aqueous suspension of Rufi nanocrystals as well aqueous suspension of Rufi and Rufi loaded *in situ* gel, reported previously. Nanometric size of the Rufi nanocrystals combined with the *in situ* gelling properties helped the optimized formulation achieve higher brain distribution and also sustain the drug concentrations in brain for longer duration compared to any of the formulations studied by our research group.

## 1 Introduction

Rufinamide (Rufi) is an anti-epileptic drug approved for the treatment of Lennox-Gastaut Syndrome (LGS) and partial seizures. LGS is a form of childhood epilepsy which manifests into adulthood and has a high morbidity and mortality rate. A general principle for therapeutic treatment of LGS is to prescribe least possible number of drugs at lowest possible doses at a time (Jahngir, Ahmad, and Jahangir, 2018). Banzel^®^, a drug product of Rufi, was first approved by the FDA in November 2008, for the adjunctive treatment of seizures associated with LGS in adults and children with ≥4 years of age. In February 2015, the United States Food and Drug Administration (FDA) approved a supplemental New Drug Application (sNDA) for Banzel^®^ as an adjunctive treatment of seizures associated with LGS in pediatric patients from 1 to 4 years of age (Eisai Inc., 2015). Orally administered Rufi shows poor oral bioavailability due to low solubility and dissolution rate in gastrointestinal fluids irrespective of the pH conditions (between pH 1.2 to 7.4) (Shorvon et al., 2015). Due to low oral bioavailability, enough drug does not reach the brain, the target organ. Therefore, Rufi is recommended to be administered at high dose in children as well as adults (maximum of 3200 mg/day in adults) and high dosing frequency through oral route to achieve the required therapeutic concentrations in brain for effective management of the disease (Epilepsy Foundation, 2022). However, administering high doses of Rufi through oral route results in aggravation of its peripheral side effects such as fatigue, rash, blistering of skin, nasopharyngitis and diplopia. Considering the shortcomings of orally administered Rufi and given that it is prescribed in pediatric patients, it is essential to look for an alternate delivery route and/or formulation for Rufi.

Nose-to-brain pathway for delivery of CNS acting drugs has been explored over the last two decades. Several reports have shown effective uptake of small molecules, biomolecules, and peptides *via* the olfactory neurons or trigeminal neurons (Jogani et al., 2008; Perez et al., 2012; Pang et al., 2016). Formulation strategies like mucoadhesive formulations, hydrogels, *in situ* gels, particulate formulations, lipid-based formulations have been used to enable and enhance the nose-to-brain uptake of therapeutic agents (Gänger and Schindowski, 2018).

Over the last decade, fabrication of nanocarriers for delivery of molecules *via* nose-to-brain pathways has gained importance. It has been observed that drug loaded nanoformulations could help in transporting the drug through the nasal mucosa by increasing the retention time at the mucosal surface resulting in increased drug concentration at the site of interest (Emad et al., 2021). As an attempt to improve the direct nose-to-brain delivery of Rufi, we prepared nanocrystal formulations of Rufi and evaluated their plasma and brain pharmacokinetics. In one of our previous publications, we prepared chitosan based nanoparticles of Rufi and suspended them in a thermoresponsive *in situ* gelling system (Dalvi et al., 2021).

As an extension to the previous work, we have formulated Rufi nanocrystals (Rufi-NCs) in this work. The aim of this work was to improve the total amount of Rufi reaching the brain when administered intranasally. Among the several nano formulations, nanocrystals have an advantage that they can carry a higher drug load than any other kind of nano formulations (Joseph and Singhvi, 2019). Nanocrystals contain near to 100% drug which is stabilized using polymers and surfactants (Wu et al., 2020). Increased dissolution rate and increased solubility are the major advantages that Nanocrystals offer over other kind of nano formulations (Gigliobianco et al., 2018). Size reduction of drug particles from micrometer to nanometer range drastically improves the surface area and in turn the rate of dissolution. Drug particles having size less than 1 μm, show a drastic change in saturation solubility of the molecule (Junghanns and Müller, 2008). Reports state that Nanocrystals with size smaller than 100 nm particles are taken up rapidly. In the nose-to-brain transport, Nanocrystals with size between 100 and 300 nm are taken up directly by the cells *via* pinocytosis while Nanocrystals with size >500 nm are taken up by phagocytosis (Bonaccorso et al., 2020).

Rufi-NCs were developed and optimized using Design of Experiments (DoE) principles. The Rufi-NCs were characterized for their particle size, zeta potential and percent yield. Rufi-NCs were suspended in previously developed xyloglucan (RXG) thermoresponsive *in situ* gel. The rheological properties of Rufi-NCs loaded *in situ* gel (Rufi-NC-RXG) was assessed to determine its sol-to-gel transition temperature and gel strength. Further, the nose-to-brain delivery Rufi-NC-RXG was compared with aqueous suspension of Rufi-NC and previously reported formulations [aqueous suspension of Rufi (Rufi-Susp) and Rufi loaded RXG *in situ* gel (Rufi-RXG)].

## 2 Materials and methods

### 2.1 Materials

Rufinamide (Rufi) and Piribedil (internal standard for HPLC method) were gift samples from Dr. Reddy’s Laboratories, Hyderabad, India. Tamarind seed xyloglucan was gifted by Encore Natural Polymers Pvt. Ltd., Ahmedabad, India. β-Galactosidase enzyme from *Aspergillus oryzae* was procured from Sigma Aldrich, Mumbai, India. Mannitol, poloxamer 407, and N-methyl–2–pyrrolidone (NMP) were purchased from Sigma-Aldrich, Mumbai, India. Hydroxypropyl methyl cellulose [HPMC E5 LV, (Methoxyl: 28.0–30.0%, Hydroxypropoxyl: 7.0–12.0%, viscosity of 2% w/v in water at 20°C is 4.0–6.0 cp)] was procured from Molychem, Mumbai, India. Thiomersal and HPLC grade solvents/chemicals such as methanol, acetonitrile, glacial acetic acid and ammonium acetate were purchased from SRL Chemicals Pvt. Ltd., Mumbai, India. Milli Q water was obtained from Millipore^®^ (MA, United States) water purification unit. Male Wistar rats were purchased from Veeba Biosciences Pvt. Ltd., Hyderabad, India.

### 2.2 Preparation of rufinamide nanocrystals

A bottom-up approach using anti-solvent precipitation technique was used to prepare Rufi-NCs. In the preliminary trials, various stabilizers and processing conditions were tried to control the crystal growth of nanocrystals. HPMC (E5 LV grade) and poloxamer 407 were selected as the stabilizers in the preparation. Briefly, 50 mg of Rufi along with poloxamer 407 (amount was varied as per the design from 5 mg to 25 mg) were dissolved in NMP (volume was varied according to the design from 1500 μl to 3000 µl). HPMC solution (percentage of HPMC solution was varied according to the design from 0.1 to 2.0% w/v) was prepared in a separate beaker. The Rufi-poloxamer solution was added to the HPMC solution using a syringe under high-speed homogenization (Polytron PT 3100D, Kinemetica, Lucerne, Switzerland). Thereafter, the homogenate was subjected to ultrasonication process (Vibra cell, Sonics, Connecticut, United States). All the processing was carried out at controlled temperature conditions. The obtained nanosuspension was centrifuged at 11,000 × g at 10°C, for 40 min. The pellet obtained was redispersed in Milli Q water containing mannitol as a cryoprotectant and freeze dried.

### 2.3 Experimental design for preparation of Rufi-NCs

Based on the literature review and preliminary trials, 8 factors (involving formulation material attributes and process parameters) affecting the critical responses of Rufi-NCs were identified. Plackett-Burman design (PBD) was used to screen the 8 factors to determine the statistically significant/critical factors affecting the responses of Rufi-NCs (Supplementary Table S1A). Each factor was studied at two different levels, which were chosen based on preliminary trials performed for the preparation of nanocrystals. The factors and their levels screened using PBD are as follows: HPMC concentration (0.1 and 2.0% w/v), amount of poloxamer (5 and 25 mg), volume of NMP (1500 and 3000 µl), processing temperature (5°C and 15°C), homogenization time (5 and 15 min), homogenization speed (7000 and 15000 rpm), ultrasonication amplitude (25% and 40%), ultrasonication time (with 3 s off and 3 s on, in pulsating mode) (5 and 15 min). The PBD consisted of 12 experimental runs plus 3 center point runs (Supplementary Table S1B). Based on the results obtained from PBD, HPMC concentration, ultrasonication time and processing temperature were found to significantly affect the responses of Rufi-NCs. Further, a high-resolution response surface design-the central composite design (CCD) was applied to understand the effect of the three critical factors on the responses and to optimize the preparation of Rufi-NCs (Supplementary Table S2 and Table 1). The polynomial equation generated from CCD is as follows:

$$Y=\beta_{0}+\beta_{1}X_{1}+\beta_{2}X_{2}+\beta_{3}X_{3}+\beta_{12}X_{1}X_{2}+\beta_{23}X_{2}X_{3}+\beta_{13}X_{1}X_{3}+\beta_{11}X_{1}^{2}+\beta_{22}X_{2}^{2}+\beta_{33}X_{3}^{2}$$ (1)

Where, Y is the response variable, $\beta_{0}$ is the mean response of all the runs given by CCD and $\beta_{i}^{\prime }s$ and $\beta_{ii}^{\prime }s$ (i=1–3) are coefficients of individual linear and quadratic effects of the factors, respectively and $\beta_{ij}^{\prime }s$ (i,j=1–3; i < j) are coefficients of the effect of interaction between $i^{th}$ and $j^{th}$ factor.

**TABLE 1:** Standard run order of the CCD experimental design given by the software with the mean particle size of Rufi-NCs obtained in each run.

Std.	HPMC concentration (X_1_) (% w/v)	Ultrasonication time (X_2_) (min)	Processing temperature (X_3_) (°C)	Particle size (Y_1_) (nm)
12	1.05	15	10	383.3
16	1.05	10	10	444.8
4	1.61	12.9	7.0	583.3
8	1.61	12.9	12.9	550.7
6	1.61	7.02	12.9	595.8
14	1.05	10	15	449.3
11	1.05	5	10	480.9
18	1.05	10	10	500.3
1	0.48	7.0	7.0	299
15	1.05	10	10	474.2
3	0.48	12.9	7.0	256.8
2	1.61	7.0	7.0	601.2
20	1.05	10	10	468.5
9	0.1	10	10	234.1
10	2	10	10	910
5	0.48	7.0	12.9	314
7	0.48	12.9	12.9	261.5
13	1.05	10	5	475.8
19	1.05	10	10	510.9
17	1.05	10	10	518.8

### 2.4 Desirability function and regression model validation

Design-Expert version 11 software (Stat-Ease Inc., Minneapolis, MN) was used to obtain screening and optimization designs in the preparation of Rufi-NCs. The optimized levels of critical factors in CCD were chosen based on the desirability function value. The regression model from the optimization design was validated by formulating *n* = 6 batches of Rufi-NCs. Particle size and % yield were determined for each batch. Wilcoxon rank sum test was used to compare the theoretical (values predicted by the model) and experimental values for particle size and % yield.

### 2.5 Preparation of Rufi-NCs loaded in reacted xyloglucan based *in situ* gel

Reacted xyloglucan (RXG) was prepared using a method published previously by our group (Dalvi et al., 2021). Briefly, tamarind seed xyloglucan solution (3.0% w/v) was prepared using 10 mM sodium acetate buffer (pH 5.0) as the solvent. This solution was reacted with β-galactosidase enzyme for 18 h at 35°C for partial removal of galactoside moieties. The reaction was terminated by heating the mixture to 90°C for 30 min. RXG was precipitated from the mixture by the addition of 95% v/v ethanol. The precipitated RXG was filtered and dried at 50°C in an oven. RXG (2.0% w/v) was dissolved in Milli Q water to form the *in situ* gel. In our earlier work (Dalvi et al., 2021), the optimization of RXG *in situ* gel was discussed in detail. Rufi-NCs loaded *in situ* gel (Rufi-NC-RXG) was prepared by dispersing the optimized, freeze dried Rufi-NCs (equivalent to the selected dose of Rufi) in 2.0% w/v solution of RXG using a magnetic stirrer. Finally, thiomersal (0.01% w/v, preservative) was added in the Rufi-NC-RXG *in situ* gel.

### 2.6 Characterization of formulations

#### 2.6.1 Measurement of particle size and zeta potential of Rufi-NCs prepared in screening and optimization designs

Zetasizer (Nano ZS, Malvern Instruments Ltd., Worcestershire, United Kingdom), which works on the principle of dynamic light scattering (DLS), was used to measure the particle size, polydispersity index (PDI), and zeta potential of the Rufi-NCs. The formulations were suspended in Milli Q water and equilibrated at 25°C for 2 min prior to the particle size measurement. The scattering intensity measurements were performed at a back scatter angle of 173°.

#### 2.6.2 Determination of % yield of Rufi-NCs prepared in screening and optimization designs

To calculate the % yield of Rufi-NCs, freshly prepared Rufi-NCs suspension was centrifuged at 10,000 × g and the supernatant was discarded. The pellet of nanocrystals was dried using a rotary evaporator (SCANVAC Scan Speed 32, Labogne ApS, Lynge, Denmark). Further, the dried pellet was dissolved in a suitable solvent and the amount of Rufi was analyzed using a HPLC method. The % yield was calculated as follows:

$$\%\;Yield=\frac{W_{total\;Rufi\;added}-W_{Rufi\;in\;pellet}}{W_{total\;Rufi\;added}}\times 100$$ (2)

Where, $W_{total\;Rufi\;added}$ is the Rufi amount used in the formulation of Rufi-NCs and $W_{Rufi\;in\;pellet}$ is the Rufi amount present in the pellet obtained after centrifugation of the Rufi-NCs suspension.

#### 2.6.3 Morphological analysis of optimized Rufi-NCs

To analyze the particle size and morphological features of the nanocrystals and bulk drug, scanning electron microscope (FE-SEM, FEI, Apreo LoVac, TermoFisher Scientific, MA, United States) was used. Around 50 µl of Rufi-NC suspension was placed on a carbon tape stuck to an aluminum stub. The Rufi-NC suspension was left under a vacuum to dry for 12 h. After complete drying, the samples, were sputter coated (Sputter Coater, Leica EM 200, Wetzlar, Germany) with a thin layer of gold in an inert gas (Argon) environment. The images were acquired at 5 kV acceleration voltage.

#### 2.6.4 Thermal analysis of optimized Rufi-NCs using differential scanning calorimeter

To check the incompatibilities between Rufi and the various excipients used in the preparation of Rufi-NC-RXG, differential scanning calorimetry analysis (DSC) (DSC 60; Shimadzu Corporation, Kyoto, Japan) was performed for nanocrystal formulations, the bulk drug, and a physical mixture consisting of all the components of the nanocrystal formulations. Around 5 mg of samples were weighed in aluminum pans and crimp sealed. Empty aluminum pan was used a reference for the analysis. The reference and test pans were placed in the DSC sample compartment and equilibrated at 25°C for 2 min before starting the run. The samples were heated at a rate of 10°C/min from 10°C to 300°C under an inert gas environment and the thermograms were recorded.

### 2.7 Rheological evaluation of optimized Rufi-NC-RXG *in situ* gel

The rheological behavior of the optimized Rufi-NC-RXG *in situ* gel was evaluated using a rheometer (Anton Paar MCR 302, Graz, Austria) to determine its sol-to-gel transition temperature and the change in elastic modulus (G′) as a function of temperature. The rheological experiments were performed in the linear viscoelastic region of the formulation. An amplitude sweep was first performed to determine the linear viscoelastic region. All the experiments were performed in triplicates.

### 2.8 Stability of optimized Rufi-NCs and Rufi-NC-RXG *in situ* gel

Freeze dried optimized Rufi-NCs were filled in air-tight vials and stored at room temperature conditions (25 ± 2°C and relative humidity of 60 ± 5%). In our previous work, we have reported that the developed RXG *in situ* gels are recommended to be stored at refrigerated conditions. Hence, the stability studies of Rufi-NC-RXG *in situ* gel were performed at refrigerated conditions (2°C–8°C). From the day of study, samples were collected on every 15^th^ day from the stored formulations. For Rufi-NCs, at each sampling point, particle size, zeta potential and percent of Rufi remaining in the formulation were determined while for Rufi-NC-RXG, sol-to-gel transition temperature and gel strength were also determined in addition to the parameters mentioned above. Stability studies were conducted in triplicates for each formulation.

### 2.9 *In vivo* studies in male Wistar rats

Male Wistar rats weighing between 240–260 g were used for all the *in vivo* studies. The *in vivo* experimental procedures were reviewed and approved prior to the studies (Approval number- BITS-Hyd/IAEC/2017/19). The formulations, Rufi-NC-RXG and Rufi-NC-Susp were administered intranasally to rats and their plasma and brain concentration vs. time profiles were constructed and compared. The animals were housed in CPCSEA approved facility. Food and water were provided *ad libitum*, except during the experimentation. On the day of *in vivo* studies, prior to dosing of the formulations, the animals were fasted for 12 h.

#### 2.9.1 Administration of formulations in rats through intranasal route

In our previously published work (Dalvi et al., 2021), we elaborated on the intricacies of intranasal dosing and its optimization in rats. A cannula-microtip device was designed for precise deposition of the formulation in the olfactory region of the rat. 10 µl of the formulation was administered using the cannula-microtip device in one nostril while the animal was still anesthetized. After dosing the formulation, the animal was kept under supine position for a couple of minutes until the anesthesia wore off.

#### 2.9.2 Dosing precision studies of Rufi-NCs and Rufi-NC-RXG formulations

The precision of dosing of cannula-microtip device was assessed in terms of % RSD of the amount of drug recovered by dispensing the formulation multiple times (*n* = 6) with the device. 10 µl of formulation (Rufi-NC-RXG or Rufi-NC-Susp) was pipetted using the dosing device and the amount of Rufi dispensed was quantified using a HPLC method.

#### 2.9.3 Assessment of mucociliary transport time of Rufi-NCs and Rufi-NC-RXG formulations

To determine the mucociliary transport time (MTT) of Rufi-NC-Susp and Rufi-NC-RXG, the rats were dosed intranasally with 10 µl of each formulation in one nostril using the cannula-microtip device. At predetermined time points, the oropharyngeal cavity of rats was wiped using a cotton swab till 360 min of dosing the formulations. Rats were restrained from having food/water till 2 h after dosing. The cotton swabs collected at every time point were treated with an appropriate solvent to extract Rufi and analysed using a HPLC method. This study was conducted in *n* = 3 rats for each formulation.

#### 2.9.4 Brain and plasma pharmacokinetic analysis of Rufi-NCs and Rufi-NC-RXG formulations

Rufi-NC-Susp and Rufi-NC-RXG were administered intranasally in male Wistar rats to determine the time course of Rufi in brain and plasma of the two formulations. The drug dose was fixed at 1 mg/kg in the study. The dosing volume of the formulations was optimized at 40 μl/kg in a single nostril. The blood was sampled at pre dose, and 5, 15, 30, 45, 60, 120, 240, 360, 480, and 600 min after dosing. Blood sampling was done by puncturing the retro-orbital plexus of rats. For the brain pharmacokinetic study, *n* = 4 rats per time point were sacrificed, and their brains were collected at 30, 60, 120, 240, and 480 min post dosing. Plasma and brain samples were processed and Rufi was quantified using methods published previously by our research group (Dalvi et al., 2021). Phoenix WinNonlin software version 8.1 was used to process the data and compute the pharmacokinetic parameters. The pharmacokinetic data in brain and plasma for Rufi-NC-RXG and Rufi-NC-Susp were compared with the data of aqueous suspension of Rufi (Rufi-Susp) and Rufi loaded RXG *in situ* gel (Rufi-RXG) taken from our previous published work) (Dalvi et al., 2021).

#### 2.9.5 Quantifying direct nose-to-brain uptake of Rufi-NCs and Rufi-NC-RXG formulations

The distribution of drug to brain following the intranasal administration of Rufi-NC-RXG and Rufi-NC-Susp was assessed by determining the % DTE (Direct transport efficiency) and % DTP (nose to brain direct transport percentage) of the formulations.

$$\%DTE=\left(\frac{{\left({AUC}_{brain}/{AUC}_{blood}\right)}_{i.n}}{{\left({AUC}_{brain}/{AUC}_{blood}\right)}_{i.v}}\right)\times 100$$ (3)

$$\%DTP=\frac{B_{i.n}-B_{x}}{B_{i.n}}\times 100$$ (4)

$$Were,B_{x}=\frac{B_{i.v}}{P_{i.v}}\times P_{i.n}$$ (5)

Where B_i.n_ is the AUC_0→tlast_ in brain of the test formulation administered through nasal route; B_i.v_ is the AUC_0→tlast_ in brain following intravenous administration of Rufi; P_i.n_ is the AUC_0→tlast_ in blood of the test formulation administered through nasal route; P_i.v_ is the AUC_0→tlast_ in blood following intravenous administration of Rufi and B_x_ is the AUC_0→tlast_ in brain contributed by the distribution of drug from systemic circulation into the blood brain barrier (BBB) following nasal administration of the test formulation. A higher % DTE value indicates higher overall transport to the brain, both *via* the direct nose-to-brain pathways and *via* nasal to systemic to brain pathway. The % DTP value indicates the percentage of transport *via* direct nose-to-brain pathways.

### 2.10 Statistical analysis

The data obtained from experimental runs of the screening design (PBD) was subjected to ANOVA to identify the critical variables. In the optimization design (CCD), the significance of regression model for each response was determined based on various diagnostic plots and the values of adjusted R^2^ and predicted R^2^. All experiments were performed in *n* = 3 replicates, and the data is presented as mean ± SD. One-way ANOVA, at α = 0.05, was used to compare the pharmacokinetic data of different formulations. Wherever required, a suitable post-hoc test was applied to compare the data to draw relevant statistical inferences.

## 3 Results

### 3.1 Preliminary trials for preparation of Rufi-NCs

Rufi-NCs were designed based on the principles of design of experiments (DoE). Particle size of nanocrystals is an important property which can affect their uptake *via* the neuronal pathways in the direct nose-to-brain delivery. Zeta potential of nano formulations affect their stability and interaction with biological membranes. Percent yield of nanocrystals is an indication of the efficiency of the manufacturing process and also it affects the amount of nanocrystals administered to get the desired dose of the drug. Hence, particle size (nm), zeta potential (mV), and % yield of the nanocrystals were taken as critical responses. A few preliminary trials were performed before applying the DoE, to choose the appropriate combination of stabilizers, solvents for dissolution of Rufi, appropriate solvent to anti-solvent ratio, and processing conditions for the preparation of Rufi-NCs using anti-solvent precipitation technique. Solubility of Rufi was checked in several organic solvents like ethanol (1:0.6), NMP (1:0.03), methanol (1:1.7), ethyl acetate (1:0.4), dimethyl formamide (DMF) (1:0.24), and acetone (1:0.33). The values in the parentheses represent the drug: solvent ratio, indicating the number of parts of solvent required to completely dissolve 1 part of Rufi. The solubility of Rufi was found to be relatively higher in NMP and DMF compared to other solvents. Further, few trials were performed with a combination of steric stabilizers (*viz.* methyl cellulose, hydroxypropyl methyl cellulose, hydroxypropyl cellulose of different viscosity grades) and amphiphilic surfactants (different grades of pluronic). The final formulation consisted of HPMC E5 LV (5 cp viscosity grade) and poloxamer 407 as stabilizers for Rufi-NCs. NMP was selected as a solvent for dissolving Rufi.

### 3.2 Screening and optimization design for preparation of Rufi-NCs

From the preliminary trials, 8 factors and their upper and lower limits were identified. A PBD screening design with resolution III was used to identify the critical factors. Out of the eight factors, three factors *viz.* factor X_1_- HPMC concentration (% w/v), factor X_2_: ultrasonication time (min), and factor X_3_: processing temperature (°C) were found to be critically affecting the responses. Hence, these factors were taken up for further optimization. An inscribed CCD was used for optimization of the three critical factors to achieve desired responses for Rufi-NCs. In the screening design (PBD), the zeta potential and % yield did not show much change with the experimental run conditions. Hence, for the optimization design only one response, particle size (nm) was evaluated. The standard run order of experimental runs of the optimization design, with the levels of each factor in their original scale, and the responses obtained are given in Table 1.

The results obtained from ANOVA of the regression model relating the particle size and factors studied in CCD are presented in Table 2. Particle size of Rufi-NCs was significantly affected by those factors with *p* < 0.05. Further, ‘F_Cal_’ values of model term, lack-of-fit and pure error were compared with corresponding ‘F_Crit_’ to determine the significance of the model. The regression equation for particle size in the coded form after deleting the insignificant factor coefficients from the model is given below:

$$Particle\;Size\;\left(Y_{1}\right)=486.73+171.08X_{1}-23.57X_{2}-30.86X_{2}^{2}$$ (6)

**TABLE 2:** Statistical output obtained from the ANOVA of regression model relating particle size with the three critical factors.

Source	Particle size (Y_1_)		
	Sum of squares	df	*p* Value
Model	4.213E^+005^	3	<0.0001
HPMC concentration (X_1_)	3.9977E^+005^	1	<0.0001
Ultrasonication time (X_2_)	7584.68	1	*0.0859
X_2_^2^	13973.18	1	0.0244
Residual	36214.35	16	<0.0001
Lack of fit	32171.45	11	0.0835
Pure error	4042.89	5	
Total	4.575E^+005^	19	

Note: *Even though the *p* value is more than 0.05, these terms are included in the model to preserve hierarchy of the model.

**FIGURE 1:**
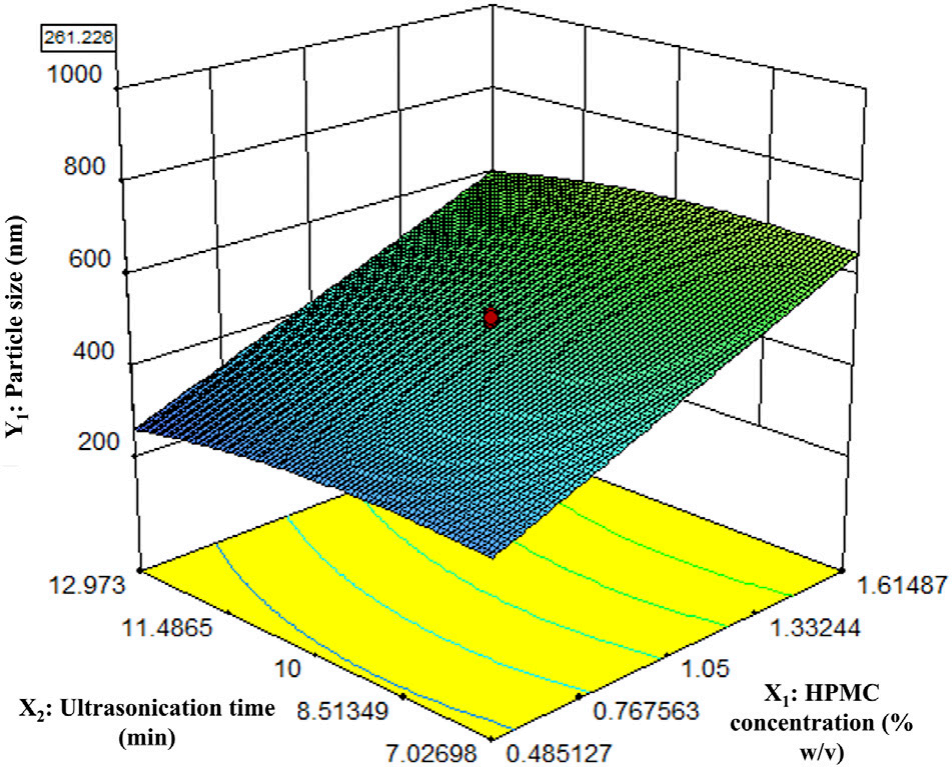
Response surface plot of particle size of Rufi-NCs as a function of HPMC concentration and Ultrasonication time.

The particle size of the nanocrystals was affected by HPMC concentration and the ultrasonication time. The response surface plot showing the effect of HPMC concentration and the ultrasonication time on the particle size of Rufi-NCs, at a given processing temperature, is presented in Figure 1. A quadratic model was found to be the best fit for defining the relationship between the particle size and the factors affecting it. The regression model was significant with ‘F_Cal_’ value of 62.04 and *p* < 0.0001. The lack-of-fit value for the model was insignificant (*p* > 0.05). The Adjusted R^2^ and Predicted R^2^ were 0.90 and 0.85, respectively. The software predicted the following experimental conditions: HPMC concentration of 0.48% w/v, processing temperature of 10°C; and ultrasonication time of 12.9 min. The optimized formulation showed a particle size of 261.2 ± 2.1 nm, PDI of 0.28 ± 0.08, and % yield of 89.6 ± 2.0. To prove the validity of the model, six replicate formulations were prepared as per the experimental conditions given by the model. The experimental and predicted values for particle size of Rufi-NCs given by the software were compared (Supplementary Table S3). No significant difference (*p* > 0.05) was observed between the experimental and predicted values.

### 3.3 Thermal analysis and scanning electron microscopy of optimized formulation of Rufi-NCs

**FIGURE 2:**
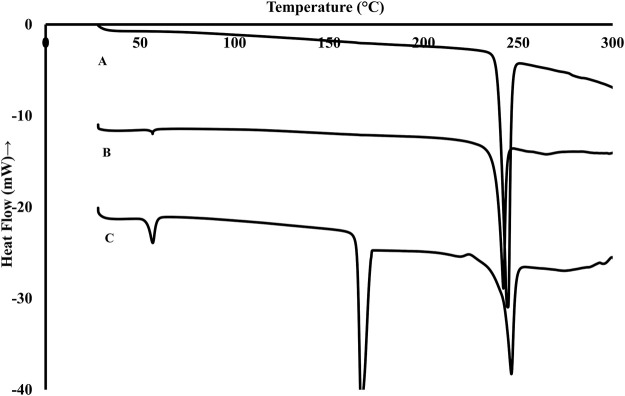
DSC thermograms of pure Rufi **(A)**, physical mixture of Rufi with all excipients used in preparation of Rufi-NCs **(B)** and Freeze dried Rufi-NCs **(C)**.

The thermograms obtained from DSC analysis of pure Rufi, physical mixture of Rufi with the excipient used in the formulation of Rufi-NCs and freeze dried Rufi-NCs are presented in Figure 2. The peak at 240°C corresponds to melting endotherm of Rufi. The physical mixture showed two endothermic peaks, one at 55°C corresponding to the melting of Poloxamer 407 and the other at 240°C for Rufi. The thermogram for Rufi-NCs showed three peaks at 55°C, 160°C, and 240°C corresponding to melting transitions of poloxamer 407, mannitol and Rufi, respectively. Mannitol was used as a cryoprotectant for freeze drying the Rufi-NCs.

**FIGURE 3:**
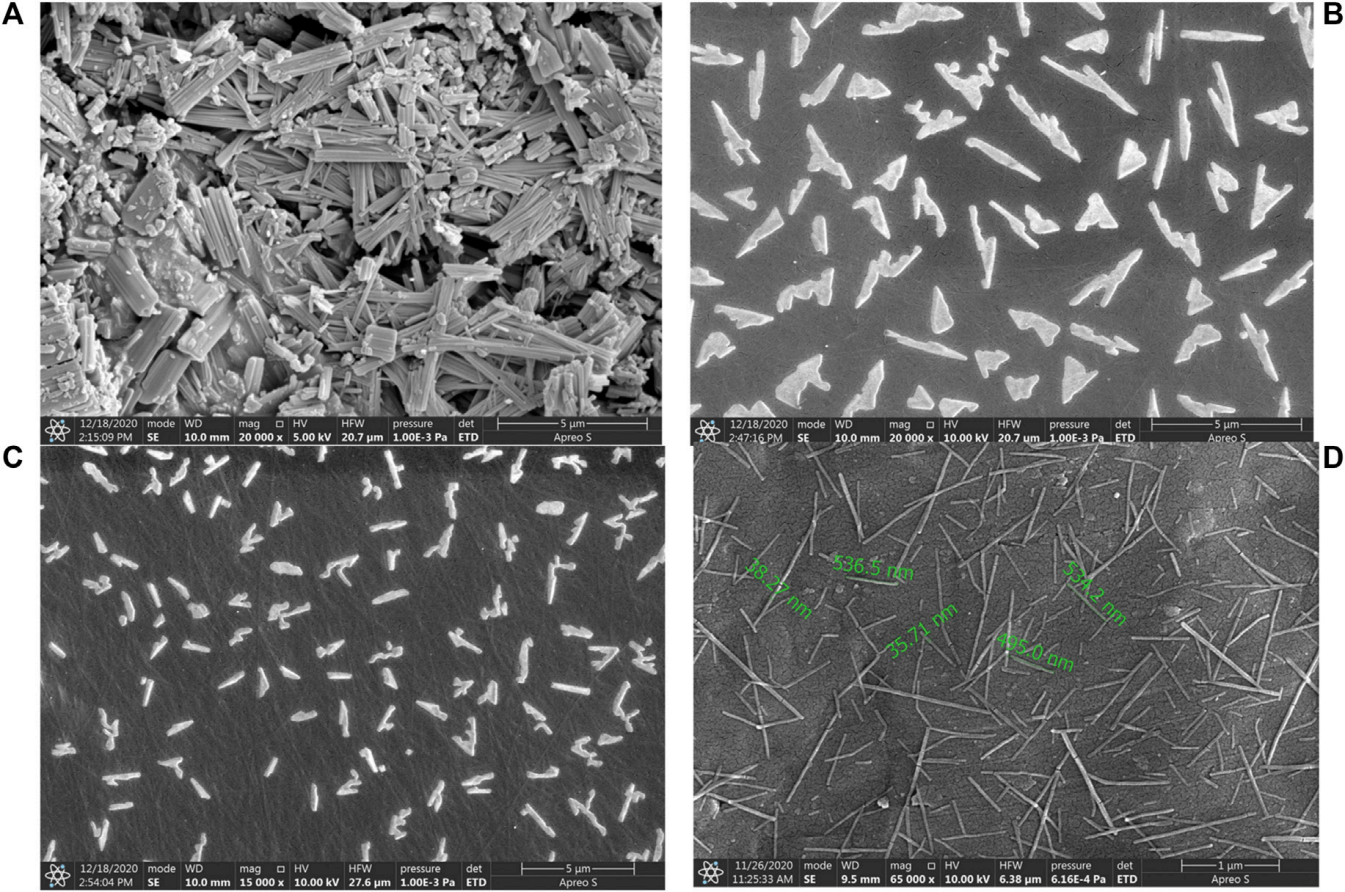
SEM image of bulk Rufi **(A)**, Rufi-NCs prepared using Standard run number 10 (particle size of 910 nm as per zeta-sizer) **(B)**, Standard run number 20 (particle size of 468 nm as per zeta-sizer) **(C)** and Standard run number 9 (particle size of 234 nm as per zeta-sizer) **(D)**.

The scanning electron microscopic images of Rufi-NCs prepared by three different standard run compositions in the optimization design (CCD) are given in Figure 3. The particle size and zeta potential of the same Rufi-NCs were also determined by zetasizer.

### 3.4 Rheological evaluation of optimized Rufi-NC-RXG *in situ* gel

**FIGURE 4:**
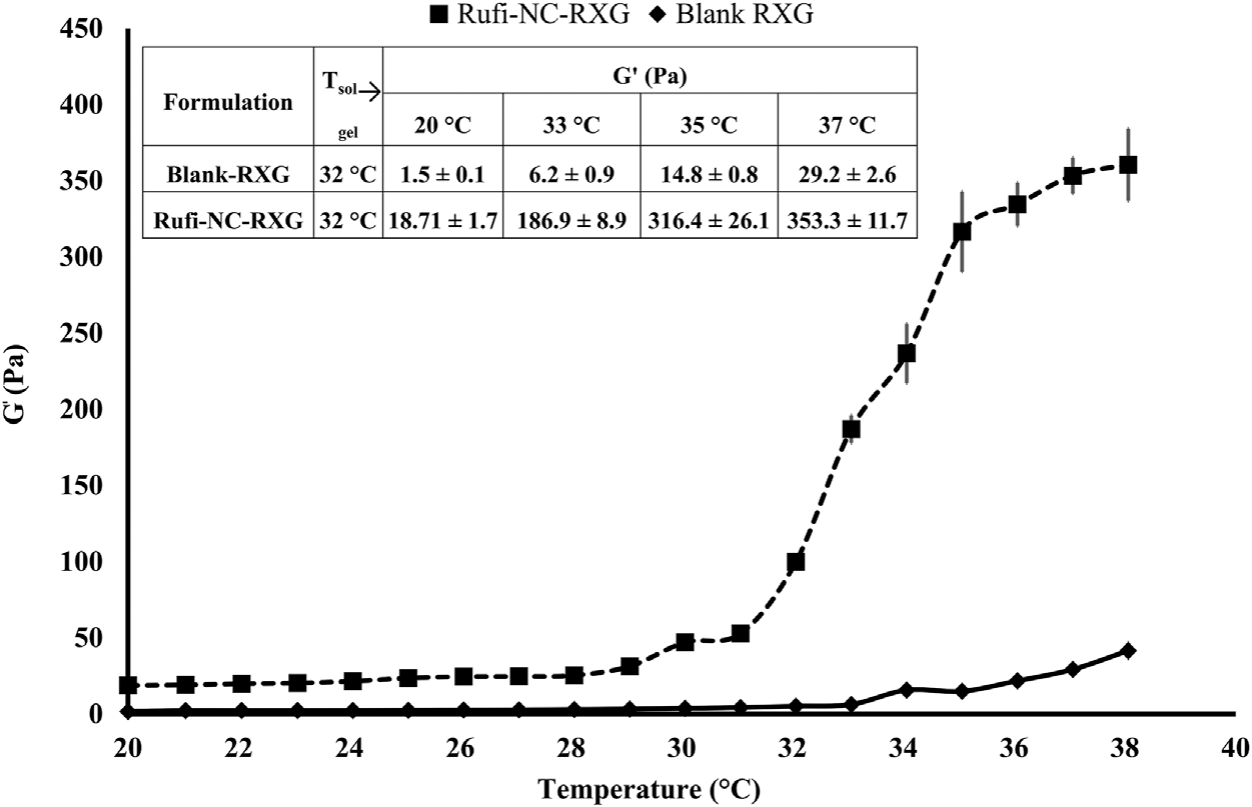
Rheological evaluation for Rufi-NC-RXG and Blank-RXG using temperature sweep.

The sol-to-gel transition temperature of Rufi-NC-RXG was compared with the Blank-RXG, reported in our previously published work. A comparative plot of change in elastic modulus (G′) vs temperature of Rufi-NC-RXG and Blank-RXG is presented in Figure 4. In the temperature range studied (20°C–38°C), the G′ values of Rufi-NC-RXG were higher than Blank-RXG at every temperature.

### 3.5 Stability of optimized Rufi-NCs and Rufi-NC-RXG *in situ* gel

The Rufi-NC-Susp and Rufi-NC-RXG formulations showed no change in the particle size and % yield values even after 60 days of storage at their respective storage conditions. The % bias values for particle size and % yield for both the formulations were found to be less than 5% at every sampling point till 60 days. Both Rufi-NC-Susp and Rufi-NC-RXG *in situ* gel exhibited good physical and chemical stability. The results obtained from stability studies are presented in Table 3.

**TABLE 3:** Stability studies of freeze dried Rufi-NCs and Rufi-NC-RXG *in situ* gel.

Stability of freeze dried Rufi-NCs (stored at 25°C and 60 ± 5% RH)					
Parameter evaluated	Time (days)				
	0	15	30	45	60
Particle size (nm)	244 ± 5.2	243 ± 7.3	244 ± 8.7	254 ± 14.3	255 ± 12.8
% Yield	87.3 ± 5.6	86.3 ± 5.5	85.6 ± 5.5	83.6 ± 4.5	83 ± 3.6
**Stability of Rufi-NC-RXG *in situ* gel stored at refrigerated condition (2°C–8°C)**					
Particle size (nm)	248 ± 6.5	243 ± 7.5	244 ± 21.5	239 ± 15.3	253 ± 6.1
% Yield	89.6 ± 2.0	86 ± 2.6	87.6 ± 2.5	85 ± 4.5	85.3 ± 5.6

### 3.6 *In vivo* studies Rufi-NC-RXG *in situ* gel in male Wistar rats

#### 3.6.1 Intranasal administration of formulations and dosing precision studies for Rufi-NCs and Rufi-NC-RXG formulations

The microtip-cannula device was used to deposit the formulations near the olfactory region of the rat’s nose. As per the literature the permissible dose volume for intranasal administration in rats is not more than 120 μl/kg (Turner et al., 2011). The volume of formulation dosed should be such that it should neither leak from the nasopalatine duct nor drain back from the animal’s nose. To identify the suitable dosing volume, rats were dosed Rufi-NC-Susp mixed with amaranth dye at different dose volumes. The nostril and the nasopalatine duct of the animals were immediately examined for leakage of the formulation by monitoring the appearance of amaranth dye. There were no signs of any leakage from the nostril and the nasopalatine duct at the minimum dosing volume of 40 μl/kg of the formulation. In addition, a dosing volume of 40 μl/kg was sufficient to deliver the required dose of the drug considering the drug content in the developed formulations.

Rufi-NC-Susp and Rufi-NC-RXG exhibited moderate viscosity at room temperature (25°C). Precise dosing for such viscous liquids with low dosing volumes can be challenging. Variability in dosing volume of the formulations can contribute to variability in the pharmacokinetic data obtained form *in vivo* studies. Therefore, prior to performing nasal pharmacokinetic studies, the precision of microtip-cannula device in dosing the nanocrystal formulations was evaluated. The % RSD values for Rufi delivered using the microtip-cannula device for both the nanocrystal formulations were less than 10% indicating that the dosing for *in vivo* studies was precise.

#### 3.6.2 Assessment of mucociliary transport time (MTT) of Rufi-NCs and Rufi-NC-RXG formulations

The MTT values of Rufi-Susp, Rufi-NC-Susp, and Rufi-NC-RXG were 11.6 ± 2.8 min, 26.6 ± 5.7 min and 43.3 ± 5.7 min, respectively. Rufi-NC-RXG showed statistically higher (*p* < 0.05) MTT value compared to Rufi-NC-Susp as well as Rufi-Susp (*p* < 0.01). The MTT values for both Rufi-NC-Susp and Rufi-NC-RXG were significantly higher than Rufi-Susp (*p* < 0.05). Higher residence time of Rufi-NC-RXG compared to Rufi-NC-Susp can be attributed to its higher gel strength (following its sol-to-gel transition upon nasal administration), which resists mucociliary clearance of formulation from the nasal cavity (Uppuluri et al., 2021a).

#### 3.6.3 Pharmacokinetic analysis and quantification of direct nose-to-brain uptake of Rufi-NCs and Rufi-NC-RXG formulations

**FIGURE 5:**
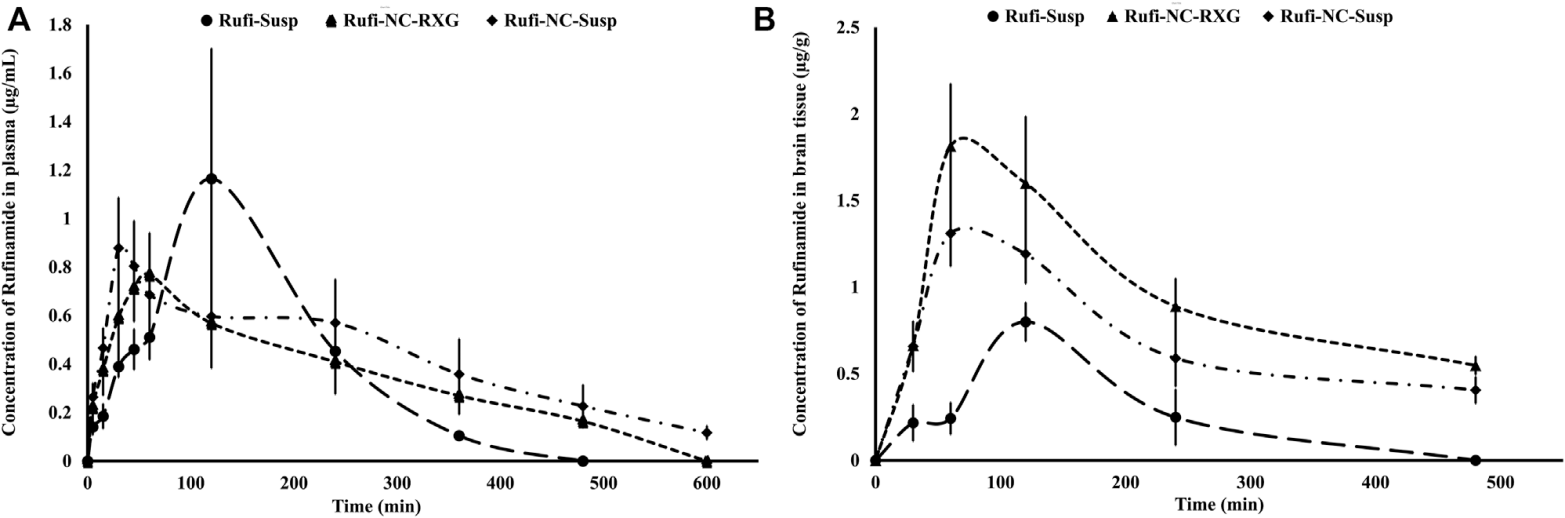
Plasma and brain pharmacokinetic performance of formulations. **(A)**: Mean concentration-time profiles of Rufi obtained following intranasal administration of Rufi-NC-Susp, Rufi-NC-RXG, and Rufi-Susp in plasma. **(B)**: Mean concentration-time profiles of Rufi obtained following intranasal administration of Rufi-NC-Susp, Rufi-NC-RXG, and Rufi-Susp in brain.

The plasma time course profiles of Rufi-NC-Susp and Rufi-NC-RXG are shown in Figure 5A. We compared the pharmacokinetic performance of Rufi-NC-Susp and Rufi-NC-RXG with that of Rufi-Susp and Rufi-RXG (Table 4) (Pharmacokinetic data for Rufi-Susp and Rufi-RXG were taken from our previously published work) (Dalvi et al., 2021).

**TABLE 4:** Pharmacokinetic parameters of Rufi-NC-RXG, Rufi-NC-Susp, Rufi-Susp, and Rufi-RXG in brain and plasma following intranasal administration at a drug dose of 1 mg/kg in male Wistrar rats.

Matrix	Parameter	Units	Formulations			
			Rufi-NC-Susp	Rufi-NC-RXG	Rufi-Susp	Rufi-RXG
Plasma	AUC_0→tlast_	Min ×( µg/ml)	258.2 ± 68.6	192.3 ± 59.5	200.75 ± 74.4	53.34 ± 10.4
	C_max_	µg/mL	0.92 ± 0.17	0.76 ± 0.17	1.16 ± 0.53	0.32 ± 0.02
	T_max_	min	30–45	60	120	120
	MRT	min	223.8 ± 9.0	177.4 ± 16.4	147.4 ± 9.4	136.3 ± 12.2
Brain	AUC_0→tlast_	Min × (µg/g)	340.7	471.3	104.28	201.8
	C_max_	µg/g	1.31 ± 0.19	1.81 ± 0.3	0.79 ± 0.11	1.48 ± 0.09
	T_max_	min	60	60	60	60
	%DTE		373.16	693.1	146.8	1069.9
	%DTP		73.3	85.5	31.9	90.6

Note: Data is represented as “mean ± SD” of *n = 4* observations.

Pharmacokinetic parameters like AUC_0→tlast_, C_max_, T_max_, and MRT were calculated by NCA (Non-compartmental analysis) using Phoenix WinNonlin Version 8.1. One-way ANOVA of the plasma AUC_0→tlast_ values of all the four formulations showed statistically significant difference between the formulations. Tukey’s HSD post-hoc test was used to find out which two groups were different from each other. The plasma AUC_0→tlast_ values of Rufi-NC-RXG and Rufi-NC-Susp were significantly higher (*p* < 0.01) than that of Rufi-RXG and Rufi-Susp. This could be due to the higher solubility and dissolution rate of Rufi-NCs in the nasal fluids, resulting in higher systemic absorption of Rufi. The plasma MRT values of both nanocrystal formulations were significantly higher (*p* < 0.01) than of Rufi-Susp and Rufi-RXG. The plasma C_max_ values of the nanocrystal formulations were not significantly different than Rufi-Susp formulations.

The pharmacokinetic parameters obtained from the brain time course of all four treatments are shown in Table 4. The brain concentrations vs time profile of Rufi-NC-Susp, Rufi-NC-RXG, and Rufi-Susp are shown in Figure 5B. At each time point for every treatment group, *n* = 4 animals were sacrificed. The concentration of Rufi at each time point was an average of the pooled concentration from 4 animals. The underlying assumption for pooling the concentration from different animals is that inter-individual differences accounted for residual variability rather than the inherent differences in pharmacokinetic process of different treatments. The average brain concentration vs. time profile could be constructed only from pooled concentration data, and hence, values of brain AUC_0→tlast_ were given as single values without standard deviation. Consequently, ANOVA test could not be applied to brain concentrations unlike plasma concentrations. The brain AUC_0→tlast_ values of Rufi-NC-Susp and Rufi-NC-RXG were almost 3 and 4.5 times the brain AUC_0→tlast_ values for Rufi-Susp, respectively. The brain AUC_0→tlast_ values for Rufi-NC-Susp and Rufi-NC-RXG were almost 1.7 and 2.3 times the brain AUC_0→tlast_ values for Rufi-RXG, respectively. The brain C_max_ values for both nanocrystal formulations were significantly higher than C_max_ values of Rufi-RXG and Rufi-Susp. The %DTE values for Rufi-NC-Susp and Rufi-NC-RXG were 373.1 and 693.1 and the %DTP values were 73.3 and 85.5, respectively.

## 4 Discussion

In this work, Rufi-NCs nanocrystals were prepared using a bottom-up approach which involved precipitation of the drug by solvent-antisolvent mixing technique. Out of the several methods reported for the preparation of nanocrystals, precipitation of the drug by solvent-antisolvent mixing is the most widely used technique (Sinha, Müller and Möschwitzer, 2013). This method uses the difference in the solubility of drug in two miscible solvents. The drug should have high solubility even at low temperatures in one solvent while the solubility of drug should be very low in the other solvent (anti-solvent). First, the drug is dissolved in the solvent in which it has good solubility to form a clear solution at room temperature. The anti-solvent is then added to the drug solution. Since both the solvents are miscible with each other, they form a homogenous mixture. But the addition of anti-solvent decreases the solubility of the drug in the solvent mixture and leads to the formation of super-saturated solution of the drug. This step marks the beginning of precipitation process of the drug. The precipitation process takes place in the following order: nucleation, followed by agglomeration and particle growth. During agglomeration and aggregation stage, the number of particles decreases, and smaller particles start adhering to the surface of larger particles (Wu and Wang, 2022). Particle size and size distribution of the nanocrystal formulations can be controlled by adjusting parameters that affect the super-saturation of the drug during the precipitation process. Process related factors such as high shear mixing of the solvent mixture or application of high energy by ultrasonication can control the super-saturation phase of the process (Sinha, Müller and Möschwitzer, 2013). Ultrasonication improves micro-mixing of the solvent and the antisolvent, thereby reducing particle growth and subsequent formation of smaller particles with narrow particle size distribution (Dalvi and Dave, 2009).

The particle size of nanocrystals can also be controlled by the addition of surfactants/stabilizers in the preparation of nanocrystals by precipitation method. Surfactants/stabilizers decrease the surface tension and increases viscosity of the solvent-antisolvent mixture. Reduced surface tension increases the nucleation rate and leads to the formation of smaller particles. The increased viscosity of the solvent-antisolvent mixture reduces the number of collisions between particles and reduces the mass transfer from the solution to the surface of already formed particles (Dalvi and Dave, 2009).

In this work, suitable solvent and antisolvent systems for preparation of Rufi-NCs were identified based on the solubility studies of Rufi in different solvents. Rufi exhibited good solubility in two solvents- NMP and DMF (from solubility data in Section 3.1). NMP shows very low acute toxicity in animals while DMF is listed as a carcinogenic solvent (Prat et al., 2013). Hence, considering the better safety profile of NMP compared to DMF, NMP was selected as the solvent. Water was selected as suitable anti-solvent as Rufi has very low solubility in it (∼30 μg/ml) (Salunkhe et al., 2018).

Literature reports state that the choice of stabilizers is entirely empirical. Khan *et al.* reported that there is no defined relationship between the drug’s physicochemical properties and the choice of stabilizer (Khan et al., 2013). Toumela et al. has also reported that the factors determining the stabilization effect of stabilizers are not fully understood and that the selection of stabilizer for drug nanocrystals is empirical (Tuomela et al., 2016). An important consideration for the choice of stabilizer is that the solubility of Rufi in the solvent-antisolvent mixture should not be affected by the stabilizer. Hence, stabilizers which did not increase the solubility of Rufi in the solvent-antisolvent mixture were tested. In this formulation, the chosen stabilizer- HPMC did not affect the solubility of Rufi in the solvent-anti solvent mixture. Further experimentation revealed that a combination of poloxamer 407 and HPMC E5 LV resulted in a lesser particle size and PDI. Therefore, in the final optimized formulation, poloxamer 407 and HPMC E5 LV were used as the stabilizers.

The morphological analysis of Rufi-NCs by scanning electron microscopy revealed that the nanocrystals were of needle/rod shape. Dynamic light scattering technique provides the size of particles in a dispersion in terms of hydrodynamic diameter, assuming that the particles in dispersion are spherical in shape. Therefore, analyzing the particle size of Rufi-NCs using zeta-sizer which works on the principle of dynamic light scattering does not give the accurate particle size as the particles are not spherical in shape. Differences were observed in the particle size analysis of Rufi-NCs by scanning electron microscopy and zetasizer. However, the trend in particle size obtained for Rufi-NCs from scanning electron microscopy correlated with that of the particle size determined using zetasizer. Such a trend was observed for each of the Rufi-NCs formulated based on the experimental runs in CCD. Hence, particle size analysis using zetasizer was considered as a reliable measure to evaluate the effect of various factors on particle size of Rufi-NCs. The regression equation relating the effect of various factors on particle size may not give the true particle size of Rufi-NCs when analyzed using scanning electron microscopy. However, the regression equation can be used to understand the direction and magnitude in which the critical factors affect the particle size of Rufi-NCs.

In the screening design, it was observed that out of the three responses, zeta potential, PDI and % yield did not vary much with the experimental run conditions of the optimization design. The low zeta potential value of the Rufi-NCs can be attributed to very little ionization of Rufi in the pH range between 1 and 10. In addition, both the stabilizers used in the preparation of Rufi-NCs are non-ionic in nature and would not contribute to the surface charge on the nanocrystals. The % yield for different experimental runs of the optimization design did not change significantly. Hence, for the optimization design, particle size was taken as the only critical response.

In the optimization design using CCD, the results obtained from ANOVA of the regression model suggest that the model is significant with insignificant lack-of-fit. The difference between Predicted R^2^ value and Adjusted R^2^ value was less than 0.2 indicating that the results predicted by the model are significant.

The response surface plot shows that as the HPMC concentration increases, the particle size increases. This could be due to increased viscosity of the solvent mixture with increase in concentration of HPMC. A greater difference in the viscosities of solvent and anti-solvent may result in poor mixing of the two phases. This could result in non-uniformity in the super-saturation achieved, giving regions with high number of rapidly growing nuclei that have higher chance of aggregation, in turn resulting in higher particle size.

From the rheological evaluation, the G′ values of Rufi-NC-RXG were consistently higher than that of Blank-RXG. This may be attributed to the high suspended solid content present in Rufi-NC-RXG. Rapid sol-to-gel transition and strength of gel produced by Rufi-NC-RXG at 32°C, proved to be beneficial in retaining the formulation in the nasal cavity for a longer time (Uppuluri et al., 2021b).

The higher MTT value for Rufi-NC-RXG compared to Rufi-NC-Susp indicates that the formulation will stay in the nose for longer time thereby allowing more drug to get absorbed (Ravi et al., 2015; Uppuluri et al., 2021a).

The ANOVA of the plasma PK data showed no significant difference between AUC_0→tlast_ values of both the nanocrystal formulations. This may be due to the enhanced solubility of Rufi-NCs and thereby greater absorption of Rufi into the systemic circulation. However, the brain AUC_0→tlast_ of Rufi-NC-RXG was approximately 1.4 and 4.5 times greater than the brain AUC_0→tlast_ of Rufi-NC-Susp and Rufi-Susp, respectively. This can be attributed to the greater residence time of Rufi-NC-RXG in the nasal cavity, thereby allowing sufficient time for Rufi to get absorbed into the brain directly. The %DTP value of Rufi-RXG was slightly higher than that of Rufi-NC-RXG, primarily due to its smaller plasma AUC_0→tlast_ value. Hence, the %DTP was higher due to the smaller denominator value (plasma AUC_0→tlast_ of Rufi-RXG) used in the calculation of the index. However, the overall brain AUC_0→tlast_ of Rufi-NC-RXG was 2.3 times the value of Rufi-RXG. In addition, the brain C_max_ value for Rufi-NC RXG was 1.3 times the value of Rufi-RXG. The brain C_max_ values of both nanocrystal formulations (Rufi-NC-Susp and Rufi-NC-RXG) were significantly higher than the Rufi-Susp formulation. The brain T_max_ values of all the formulations was 60 min. Higher brain AUC_0→tlast_ and C_max_ values of the nanocrystal formulations could be due to the higher solubility of Rufi-NCs in the nasal fluids as well as possible direct uptake process of the nanocrystals into the brain by pinocytosis from the olfactory region of the nasal cavity.

## 5 Conclusion

In the current research work, Rufi-NCs were prepared using anti-solvent precipitation technique. Using Design of experiments, factors such as ultrasonication time, temperature during the homogenization process, and the amount of stabilizer used were identified as critical factors affecting the particle size and % yield of the Rufi-NCs. The optimized Rufi-NCs were dispersed in reacted xyloglucan to form thermoresponsive nasal *in situ* gel (Rufi-NC-RXG). The sol-to-gel transition temperature of Rufi-NC-RXG was found to be 32°C. The strength of the gel formed by Rufi-NC-RXG increased the residence time of the formulation by resisting the mucociliary clearance. Comparative nasal pharmacokinetic studies of optimized Rufi-NC-Susp and optimized Rufi-NC-RXG were performed in Wistar rats for evaluating their direct nose-to-brain delivery of Rufi. The data obtained was also compared with the earlier published data of Rufi-Susp and Rufi-RXG formulations. The pharmacokinetic parameters obtained in brain revealed that Rufi-NC-RXG showed a significantly higher (1.4 folds) direct nose-to-brain delivery of Rufi compared to Rufi-NC-Susp. Similarly, the nose-to-brain delivery of Rufi by Rufi-NC-RXG was 4.5 folds and 2.3 folds higher than Rufi-Susp and Rufi-RXG formulations, respectively. The results obtained from this work suggest that nasal administration of both Rufi-NCs formulations (Rufi-NC-Susp and Rufi-NC-RXG) would be beneficial for enhancing the distribution of Rufi to brain.

## Data Availability

The raw data supporting the conclusions of this article will be made available by the authors, without undue reservation.
