# Supplementary material for: Design and evaluation of rufinamide nanocrystals loaded thermoresponsive nasal in situ gelling system for improved drug distribution to brain
Source: Front Pharmacol. 2022 Oct 4;13:943772. doi: 10.3389/fphar.2022.943772 (PMC9577085; doi:10.3389/fphar.2022.943772)
Supplement: Supplementary file 1 [file DataSheet1.docx]

***Supplementary Material***

**Supplementary Table 1a: Factors and their levels selected for screening using Placket- Burman for formulation of Rufi-NCs**

| **Factor Code** | **Actual factors** | **Levels** | | |
| --- | --- | --- | --- | --- |
|  |  | **-1** | **0** | **+1** |
| $\boldsymbol{X}_{\mathbf{1}}$ | HPMC concentration (% w/v) | 0.1 | 1.05 | 2.0 |
| $\boldsymbol{X}_{\mathbf{2}}$ | Ultrasonication time (min) | 5 | 10 | 15 |
| $\boldsymbol{X}_{\mathbf{3}}$ | Processing temperature (°C) | 5 | 10 | 15 |
| $\boldsymbol{X}_{\mathbf{4}}$ | Amount of poloxamer (mg) | 5 | 15 | 25 |
| $\boldsymbol{X}_{\mathbf{5}}$ | Homogenization time (min) | 5 | 10 | 15 |
| $\boldsymbol{X}_{\mathbf{6}}$ | Homogenization speed (rpm) | 7000 | 11000 | 15000 |
| $\boldsymbol{X}_{\mathbf{7}}$ | Ultrasonication amplitude (%) | 25 | 32.5 | 40 |
| $\boldsymbol{X}_{\mathbf{8}}$ | Volume of NMP (µL) | 1500 | 2250 | 3000 |

**Supplementary Table 1b: Randomized run order of the Placket- Burman (PBD) experimental design for Rufi-NCs**

| Std | HPMC concentration (% w/v) | Amount of poloxamer (mg) | Volume of NMP (µL) | Processing temperature (°C) | Homogenization time (min) | Homogenization speed (rpm) | Ultrasonication amplitude (%) | Ultrasonication time (min) | Particle size (nm) | Zeta potential (mV) | Yield (%) |
| --- | --- | --- | --- | --- | --- | --- | --- | --- | --- | --- | --- |
| 12 | 0.1 | 5 | 1500 | 5 | 5 | 7000 | 25 | 5 | 186 | -1.1 | 90.8 |
| 8 | 2 | 25 | 1500 | 5 | 5 | 15000 | 25 | 15 | 700 | -1.04 | 85.2 |
| 3 | 2 | 5 | 3000 | 15 | 5 | 15000 | 40 | 15 | 1200 | 1.1 | 93.8 |
| 2 | 0.1 | 25 | 3000 | 5 | 15 | 15000 | 40 | 5 | 230.6 | -1.5 | 85.3 |
| 1 | 2 | 25 | 1500 | 15 | 15 | 15000 | 25 | 5 | 1900 | -1.2 | 95.27 |
| 5 | 0.1 | 5 | 3000 | 5 | 15 | 15000 | 25 | 15 | 162.8 | 1.7 | 86.9 |
| 4 | 0.1 | 25 | 1500 | 15 | 15 | 7000 | 40 | 15 | 225 | 1.3 | 86.39 |
| 15 | 1.05 | 15 | 2250 | 10 | 10 | 11000 | 32.5 | 10 | 539.9 | 1.4 | 90.4 |
| 14 | 1.05 | 15 | 2250 | 10 | 10 | 11000 | 32.5 | 10 | 522.9 | -1.2 | 90 |
| 6 | 0.1 | 5 | 1500 | 15 | 5 | 15000 | 40 | 5 | 175.6 | -1.7 | 92.7 |
| 13 | 1.05 | 15 | 2250 | 10 | 10 | 11000 | 32.5 | 10 | 549.6 | 1.01 | 91.9 |
| 9 | 2 | 25 | 3000 | 5 | 5 | 7000 | 40 | 5 | 1400 | 1.2 | 85.5 |
| 11 | 2 | 5 | 3000 | 15 | 15 | 7000 | 25 | 5 | 1620 | -1.07 | 91.05 |
| 10 | 0.1 | 25 | 3000 | 15 | 5 | 7000 | 25 | 15 | 223.6 | 1.34 | 87.8 |
| 7 | 2 | 5 | 1500 | 5 | 15 | 7000 | 40 | 15 | 810.6 | -1 | 93.3 |

Supplementary Table 2. Factors and their levels selected for optimization of Rufi-NCs using Central Composite Design (CCD)

| Factor Code | Actual factors | Levels | | |
| --- | --- | --- | --- | --- |
|  |  | -1 | 0 | +1 |
| A | HPMC concentration (% w/v) | 0.1 | 1.05 | 2.0 |
| B | Ultrasonication time (min) | 5 | 10 | 15 |
| C | Processing temperature (°C) | 5 | 10 | 15 |

**Supplementary Table 3: Predicted and experimental values for the response (particle size) obtained from the model**

| S. No | Model Predicted values | Experimental/observed values |
| --- | --- | --- |
| 1 | 290.1 | 262.0 |
| 2 | 284.6 | 263.4 |
| 3 | 259.9 | 258.5 |
| 4 | 306.8 | 261.3 |
| 5 | 275.3 | 263.1 |
| 6 | 250.5 | 258.8 |
